# Exploring the impact of specialist and generalist stars on organizational performance

**DOI:** 10.1371/journal.pone.0349682

**Published:** 2026-05-28

**Authors:** Georgios Nalbantis, Christian Manger, Tim Pawlowski, Philip Yang

**Affiliations:** 1 Faculty of Economics and Social Sciences, Institute of Sports Science, University of Tuebingen, Tuebingen, Germany; 2 Faculty of Economics and Social Sciences, School of Business and Economics, University of Tuebingen, Tuebingen, Germany; 3 LEAD Graduate School and Research Network in Tuebingen, Tuebingen, Germany; 4 Interfaculty Research Institute for Sports and Physical Activity in Tuebingen, Tuebingen, Germany; 5 Faculty of Business Administration and Economics, University of Paderborn, Paderborn, Germany; Guangxi Normal University, CHINA

## Abstract

We analyze the impact of specialist and generalist stars on organizational performance by linking the literature on star employees and specialization. Our adaptive role framework allows stars to switch roles over time. We develop a game-theoretical model to derive hypotheses and to empirically test them using basketball data. Consistent with theoretical predictions, the results show that teams with generalist stars outperform those with specialists. While a generalist switching to a specialist role *always* worsens performance, a specialist switching to a generalist role *may* improve performance. Additional estimations suggest that these findings hold for teams relying on a unique star rather than multiple stars.

## Introduction

Human capital is widely regarded as a critical driver of organizational productivity and success [1]. Among the most influential contributors to firm outcomes are star employees—individuals with exceptional performance, visibility, and valuable human and social capital [2]. Although extensive research has explored the benefits of recruiting stars (e.g., [3,4]), less attention has been paid to how differences in the individual qualities of stars influence organizational performance [5]. Specifically, the interplay between generalist and specialist stars remains underexplored, despite evidence suggesting that these distinctions can profoundly shape team dynamics and outcomes [6–8].

The distinction between generalists and specialists is a recurring theme in both academic (e.g., [7,9,10]) and non-academic literature. Generalists possess broad knowledge and skills, enabling them to engage in a variety of tasks, while specialists excel in specific areas but may struggle outside their domains of expertise [11]. Empirical studies offer conflicting evidence about which type is more beneficial: some suggest that generalists enhance team flexibility and creativity, especially in dynamic environments [12,13], while others indicate that high task interdependency favors specialists [14]. This ongoing debate has also extended to corporate leadership, where generalist CEOs often receive a pay premium despite mixed evidence regarding their performance impact [10,11,15]. However, the literature remains largely silent on the role of generalist and specialist *non-executive* star employees in team environments. Moreover, there have not been any attempts yet to develop and empirical test a formal theoretical model about how differences in individual star qualities relate to team performance. This paper seeks to address these gaps by examining the impact of generalist and specialist stars on team performance in the context of professional basketball.

Professional basketball provides a unique setting to study the interplay between generalist and specialist stars due to its high task interdependence and the prominent role of individual contributions in team success. Specifically, we make three key contributions: *First*, we connect research on the impact of stars on performance with literature on specialization and diversification. Following Kehoe, Lepek and Bentley [5] several types of stars exist (e.g., universal, status, networking stars). In this paper the spotlight is put on performance stars (subsequently referred to as star performers), that is, individuals who demonstrate exceptional task performance. We define stars’ generalist or specialist orientation based on their multitasking capacity, measured through their offensive play types. Following Buser and Peter [16] multitasking is defined as the switching between multiple contingent tasks. Multitasking is increasingly recognized as a critical skill, particularly in complex and uncertain environments [17]. *Second*, our analysis centers on star performers who excel in team environments, rather than executive roles, to explore their unique contributions to performance. *Third*, drawing on leadership theory, and in particular on leader flexibility, effective actors perceive the situation, generate options, and adjust their roles as conditions change [18,19]. Accordingly, unlike prior studies that treat specialization or generalization as fixed traits, we adopt an adaptive role framework in which a star’s degree of diversification can vary over time in response to organizational needs. Framing roles as adjustable may help reconcile conflicting findings in the literature by emphasizing the dynamic interplay between individual roles and team performance [20–24].

The backbone of our study is a game-theoretical model that explores the relationship between generalist and specialist stars. This model illustrates the two opposing effects of specialization on team performance: While specialist star performers allow teams to focus on few play types in which they excel, they also make a team more predictable for the opposing defense. Simulations based on the model suggest that teams with generalist stars tend to outperform those with specialist stars. Additionally, our model predicts that role-switching impacts performance asymmetrically: a generalist shifting toward specialization reduces team performance, while a specialist shifting toward generalization may enhance it. We test these predictions empirically using longitudinal game-level data from the National Basketball Association (NBA) spanning four seasons (2012/13–2015/16). Our analysis seeks to provide initial evidence supporting the theoretical model, focusing on identifying patterns and correlations rather than establishing causal relationships. By analyzing offensive play types for each player, we provide a granular measure of diversification and identify star performers. Our findings align with the theoretical predictions, underscoring the nuanced trade-offs between generalist and specialist contributions to team success. This initial supportive evidence highlights directions for future research.

The remainder of the paper is organized as follows: Section “A Simple (theoretical) game of Basketball” introduces the theoretical model, numerical simulations, and hypotheses. Section “Empirical test” details the empirical framework, including data, measures, and econometric specification. Section “Results” presents the main results, and section “Discussion and conclusion” concludes with implications and limitations.

## A Simple (theoretical) game of basketball

We use a simple game-theoretical model of a basketball game to illustrate how different types of star performers may affect the performance of teams. We focus on star performers’ multitasking capacity to derive their degree of diversification/specialization. Following Buser and Peter [16], we define multitasking as the switch between several challenging and ongoing tasks. In our setting, the play types constitute such tasks. First, we discuss how the skills of individual players in various play types determine aggregate team skills for those play types. Next, we show how these team skills, together with the strategies of both teams, determine the scoring probability. In the standard Nash equilibrium, both teams choose a strategy that maximizes their individual probability to win the game. We vary the team composition and the different types of star performers in order to infer the effect of star diversification on performance. The model illustrates the two opposing effects of specialization: Specialization allows a team to make use of complementarities in their individual skills and to focus on strong play types, but also makes the team more predictable for the defense of the opposing team.

### Individual and aggregate skills

In our game, the team that is in possession of the ball and attacks is playing offense. The other team that tries to prevent the offensive team from scoring is playing defense. Each team consists of five basketball players that act as a unit.

*Offense.* Consider a team of five basketball players, identified by n∈{1,2,3,4,5}. There are *P* different offensive *play types* such as “Transition”, “Pick and Roll” and so on, which we indicate by p∈{1,2,...,P} (for variable description see S1 Table, for play type description S2 Table). Every player has an individual skill level strictly between 0 and 1 in each of the play types, denoted by *s*_*n*,*p*_. We normalize the individual skill to $s_{n,p}\in (0,1)$. Next, we aggregate the individual skills for play type *p*, *s*_*n*,*p*_, to a “team skill” in play type *p*, *S*_*p*_. Considering the strong complementarity in individual skills for the ultimate goal (scoring), we use the O-Ring production function introduced by Kremer [25]. The O-Ring production function is commonly used in development economics as well as in a broader context in labor economics. Moreover, it has already been used in sports settings [26]: In order to successfully conduct a particular task as a group, every group member must successfully perform their individual contribution, determined by the individual skill. Thus, total success probability, or, in our case, the team skill *S*_*p*_ of the team in a particular play type *p*, is the product of all individual skills:


$$\begin{array}{c} S_{p}=\Pi_{n=1}^{5}s_{n,p}. \end{array}$$ (1)

If we later combine this offensive team skill with the defensive team skill of the opponent and both teams’ strategies, we can determine the scoring probability of this play type and overall team performance. Thus, the aggregation of individual skills to a team skill allows us to shift our focus from individual performance to the effect of the team composition and the choice of play types on the performance of the whole team.

*Defense.* As in our NBA data (see Section “Empirical test”), the theoretical model differentiates between the *P* different offensive skills corresponding to the different play types for each individual player and, in the aggregate, for each team. This focus on the offensive play types is also reflected in the model’s approach to defense: We assume there is only one general defensive skill for every player, *d*_*n*_. Thus, the aggregate general defensive skill of a team is


$$\begin{array}{c} D=\Pi_{n=1}^{5}d_{n}. \end{array}$$ (2)

In the Nash equilibrium, the team in defense has the option to focus its defensive efforts on particular offensive play types. This allows us to consider the optimal reaction of the defense without explicitly modeling multiple defensive play types.

### Optimal strategies

*Offense.* The team in offense tries to find the optimal mixed strategy that maximizes its scoring probability. Let $\sigma =(\sigma_{1},\sigma_{2},...,\sigma_{P})$ denote the offensive strategy, where $\sigma_{p}$ denotes the probability that the offensive team chooses play type *p*, given the strategy of the team playing defense (see below).

*Defense.* In general, the team in offense has an incentive to focus on the play type with the highest team skill. In the NBA, coaches and players of the defensive team will anticipate this offensive strategy σ as a function of the offensive team’s skills and can draw conclusion from previously observed play types. In our Nash equilibrium, the defensive team plays the best response against $\sigma =(\sigma_{1},\sigma_{2},...,\sigma_{P})$. We allow the team in defense to adjust their strategy to focus on defending against one or several particular play types at the cost of neglecting defense against the remaining play types. If, for instance, the team in offense is excellent at post-up plays, the defense could try to deny a crucial offensive player on the opposing team the ball or make sure a second defensive player is close enough to double-team that particular offensive player (“have someone else beat you”).

A defensive team that focuses on defending against a particular offensive play type receives a bonus when the offensive team actually chooses this play type, but receives a penalty if the offensive team chooses another play type that is now less the focus of the defense. We use $\delta_{p}$ to denote the relative weight the defense puts on defending against play type *p*.

These weights of defensive focus are used to modify the general defensive skill *D* against the different play types. In particular, the modified defensive skill against play type *p* is $D_{p}=D\sqrt{\delta_{p}P}$. If the defense puts identical attention on all offensive plays, this approach makes sure that $D_{p}=D$ for all play types. If the defense increases $\delta_{p}$ for some particular play type *p*, then the modified defensive skill increases as $dD_{p}/d\delta_{p}>0$. However, as *D*_*P*_ is concave in $\delta_{p}$, the positive effect of an increase in $\delta_{p}$ diminishes with further increases. Moreover, concavity also ensures that defensive teams never entirely focus on defending against one particular play type (that is, $\delta_{p}\neq 1$ for all *p*). While the offensive team will focus on its strongest play type but still mix its strategy so as not to be perfectly predictable, the defensive team will put more focus on defending against the strongest play type but still devote some attention to other play types. This approach ensures that it is always optimal for the offense to put more weight on its stronger play type, but it is never optimal to play *only* the strongest play type. In the same way, it is never optimal for the defensive team to focus entirely on defending against one particular play type.

The extent of the diversity of the strategies will depend on the skill composition of the offensive team and their star performer. A team playing offense with a more specialized star performer will have a less diversified strategy and focus on its particularly strong play type. However, this will also allow the defense to focus its defense on this particular play type, counteracting the benefit of specialization. Therefore, ex-ante, the effect of specialization on team performance is ambiguous.

*Scoring probability.* Consider an offensive team that chooses a particular play type *p* with team skill *S*_*p*_ and is defended by another team with defensive skill *D* and defensive focus $\delta_{p}$ against this play type. We now translate these aggregate skills (which are both between 0 and 1) into a scoring probability. We assume that the scoring probability in this scenario is


$$\begin{array}{c} \varphi (S_{p},D,\delta_{p})=\exp\left[-\frac{D\sqrt{\delta_{p}P}}{S_{p}}\right] \end{array}$$ (3)

The defensive team skill *D* is weighted by the modifier $\sqrt{\delta_{p}P}$ and then compared with the offensive team skill *S*_*p*_. This scoring probability for play type *p* is increasing in the offensive team skill *S*_*p*_, decreasing in the general defensive team skill *D*, and decreasing in the defensive focus against this play type $\delta_{p}$.

*Payoff and equilibrium.* The offensive team wants to maximize its expected scoring probability, while the defensive team wants to minimize the scoring probability. The model is solved by the Nash equilibrium in mixed strategies (σ,δ) in which both teams act rationally and maximize their individual payoffs. That is, the team playing offense will choose $\sigma =(\sigma_{1},\sigma_{2},...,\sigma_{P})$ in order to maximize its scoring probability (and thereby also its probability of winning), given the strategy δ of the team playing defense. The team that is playing defense chooses $\delta =(\delta_{1},\delta_{2},...,\delta_{P})$ such that the scoring probability is minimized for a given σ, also maximizing its own probability to win the game (A detailed discussion of the Nash equilibrium can be found in S1 File).

In this setup, the effects of a star player on a team’s performance depend on the type of the star player and the team in which he is playing. A specialist star player gives the team a significant advantage in one particular play type while not exceeding regular players in other play types. Due to complementarity in the production function, this disproportionately increases the performance of a team in which the other players also excel at the same play type. This team will find it optimal to put a strong focus on this offensive play type but also allow the defense to focus on defending against this play type. In contrast, a generalist star player increases the team’s skills for all play types moderately. This is more beneficial for teams with a more balanced skill set, allowing the team to mix well between play types and to prevent it from being too predictable for the defense.

Thus, switching from a generalist star player to a specialist star player has two opposing effects on the team performance: First, the offense with the specialist star player will play the play type more often in which the specialist excels. *For a given defensive strategy*, the performance of the offensive team that switches from the generalist to the specialist star improves. However, this lower diversification in the offense allows the defense to focus more on defending against this particular play type. *The defense’s adjustment to a less diversified strategy* reduces the performance of the offensive team that switches to a specialist star. This model aims to illustrate these two opposing effects of specialization.

While a generalist and a specialist star performer should be different in their skill distribution, they should still be “comparable”. In other words, we could always generate a superior (inferior) specialist star performer by simply increasing (decreasing) his skill in one play type until his performance is sufficiently high (low) to exceed (fall below) that of the team with a generalist. However, for our simulation, no type of star performer should have an ex-ante advantage over the other. In contrast, the differences in team performance with different types of star players must come from the interaction of the star performer’s skills with his team and the resulting choice of play types. Therefore, we rely on extensive numerical simulations with carefully constructed star performers who differ in the degree of specialization of their skills but are overall comparable in quality. These different star performers play in a variety of different team setups to examine the impact of star performers and the opposing effects of diversification of play types (benefits of specialization vs predictability) on team performance (Mathematica code and further information are available upon request).

### Simulation and results

To keep things simple and without loss of generality, we focus on three play types (*P* = 3). Moreover, we assume that in a team without a star performer, all players are identical and we denote their individual skill in play type *p* by $s_{p}^{t}$. All defensive players have identical individual defensive skills $d^{t}=0.7$.

*Baseline teams.* In order to analyze how different types of star performers affect the optimal strategy and team performance, we define various baseline teams (without star performer) that range from very balanced teams (symmetric distribution of skills over the 3 play types) to more specialized teams (asymmetric distribution of skills over play types). In particular, our first representative team (without a star performer) is perfectly balanced with $s_{1}^{t}=s_{2}^{t}=s_{3}^{t}=0.7$. This is the grey shaded team in Table 1. If this team is now playing the game without a star performer, it will score with a probability of 36.79%. This scoring probability is determined by the combination of the individual skills, the optimal offensive strategy σ and the optimal defensive weights $\delta_{p}$, evaluated by the scoring function (3).

**Table 1:** Summary of the 11 baseline teams.

$s_{1}^{t}$	0.588	0.604	0.62	0.636	0.652	0.668	0.684	0.7	0.716	0.732	0.748
$s_{2}^{t}$	0.7	0.7	0.7	0.7	0.7	0.7	0.7	0.7	0.7	0.7	0.7
$s_{3}^{t}$	0.743	0.741	0.738	0.734	0.729	0.723	0.713	0.7	0.68	0.644	0.527

Balanced (middle) and less balanced (left and right) teams with a scoring probability of 36.79% without star performers. This Table summarizes the 11 baseline teams (without star performers) which differ by play type specific skill levels. The fully balanced team is grey shaded (with $s_{1}=s_{2}=s_{3}=0.7$). Teams on the left hand side are better in skill *s*_3_ (but worse in skill *s*_1_). Teams on the right hand side are better in skill *s*_1_ (but worse in skill *s*_3_). Importantly, skill *s*_2_ is the same across all teams and all teams have exactly the same team specific scoring probability of 36.79%. A description of the variables used in the theoretical model is provided in S1 Table.

Now we make the team a bit more imbalanced by raising $s_{1}^{t}$ to $s_{1}^{t}=0.716$ while leaving $s_{2}^{t}$ unchanged at $s_{2}^{t}=0.7$. We then compute the skill for the third play type $s_{3}^{t}$ such that the performance of this slightly less balanced team is identical to the perfectly balanced one, that is, it has a scoring probability of 36.79%. This is the case for $s_{3}^{t}=0.68$. We repeat this process for different values of $s_{1}^{t}$ and $s_{3}^{t}$ until we have in total 11 different teams with varying specialization in play type 1 or 3. Table 1 summarizes these 11 baseline teams. Each of these teams without a star performer is defined by its $s_{1}^{t}$, $s_{2}^{t}=0.7$ and the level of $s_{3}^{t}$ that keeps the scoring probability at 36.79%. Thus, by construction, there is no correlation between the specialization of a team (excluding the star performer) and team performance.

*Star performers.* We now define two different types of star performers, a “generalist” and a “specialist”. We assume that in teams in which any type of star performer is present, the star performer will be player 1, while the other players 2–5 are still the players described in Table 1. The generalist star performer is always perfectly balanced with $s_{1,1}=s_{1,2}=s_{1,3}=0.8$, while the specialist is superior in the first play type, but only average in the other two, $s_{2}=s_{3}=0.7$. If we use a theoretical model to compare the performance of a generalist and a specialist, they are supposed to differ in their skill distribution, but they should be equivalent in overall quality *so as not to give one type of star performer a particular ex-ante advantage*. Thus, we choose *s*_3_ for the specialist such that in a 1 vs 1 game, both star performers are equally likely to win. The only difference between this 1 vs 1 game and the previously described (full) game is that we focus on the individual strengths of the star performers, while potential interaction effects with team members do not play any role. This approach gives rise to *s*_3_ = 0.9695 for the specialist so as not to give any type of star performer an ex-ante advantage over the other type. Any difference in team performance between teams with a generalist star performer and with a specialist star performer must come from the interaction between the star performer, his team, and the effect on the optimal play types. Table 2 shows the parametrization of the model. As a robustness check, we later also consider a specialist with the “opposite” focus, that is, $s_{1}=s_{2}=0.7$ and *s*_3_ = 0.9695 (see S1 Fig).

**Table 2:** Parameters used in the numerical simulation.

**Team**				
Parameter	$s_{1}^{t}$	$s_{2}^{t}$	$s_{3}^{t}$	*d* ^*t*^
Value	0,588–0,748	0,7	0,743 to 0.527	0,7
**Star performer (Generalist)**				
Parameter	*s*_1,1_	*s*_1,2_	*s*_1,3_	
Value	0.8	0.8	0.8	
**Star performer (Specialist)**				
Parameter	*s*_1,1_	*s*_1,2_	*s*_1,3_	
Value	0.9695	0.7	0.7	

A description of the variables used in the theoretical model is provided in S1 Table.

**Fig 1:**
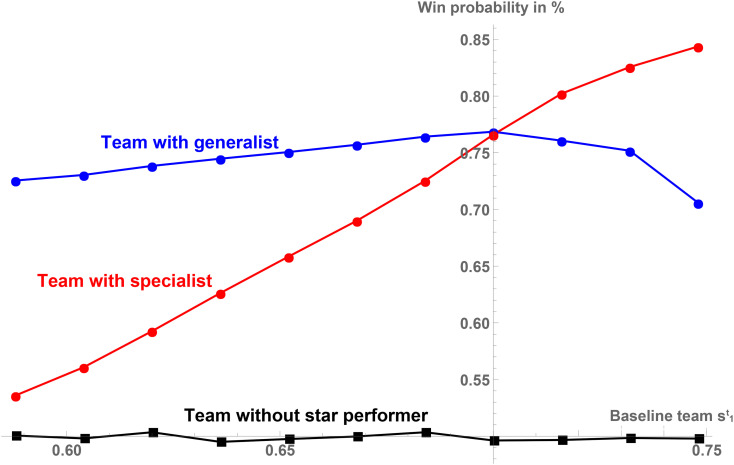
Absolute team performance for the different baseline teams. A description of the variables used in the theoretical model is provided in S1 Table. As robustness check, a relative performance measure is presented in S2 Fig.

*Simulation.* For each of the 11 baseline teams from Table 2 we simulate 20,000 games against a fixed type of opponent with $s_{p}^{t}=d^{t}=0.7$. In each game, both teams choose the optimal strategies according to our Nash equilibrium and play offense 100 times and defense 100 times. If, after these 100 plays for each team the score is tied, we simulate “overtime” by adding 10 more plays for both teams until there is a winner. In order to focus on the effects of the presence and the type of the star performer, and in line with our empirical approach, these games are independent of each other and we abstain from simulating dynamic learning or team chemistry building. Fig 1 shows the absolute performance of a team as the percentage of games that are won by the team under consideration. Note that while we focus on absolute performance, we further check the robustness of our estimations by using a relative performance measure (these results are presented in S2 and S3 Figs).

The diagram illustrates the simulated performance of the 11 different teams without a star performer (black squares), with a generalist star performer (blue circles) and with a specialist star performer (red circles). Without the star performer, there is no correlation between the different baseline teams and their performance. In particular, generalist teams ($s_{1}^{t}=0.7$) perform as well as teams specialized in play type 1 ($s_{1}^{t}>0.7$) or play type 3 ($s_{1}^{t}<0.7$) when the star is excluded. Thus, any differences in team performance are generated by adding a generalist or specialist star performer. By switching the type of star player from a generalist to a specialist (and vice versa) for a given baseline team, we can study the effect of role switching on team performance.

If we now consider teams in which player 1 is a star performer, their performance benefits significantly from both types of stars. While a generalist star is slightly more beneficial for generalist teams ($s_{1}^{t}=0.7$) than for more specialized teams (that is, baseline teams more to the left or to the right), the quantitative effect of the “fit” between a generalist and the different baseline teams seems to be negligible. A specialist star is best utilized in a baseline team that is heavily specialized toward the strength of the star player in play type 1 ($s_{1}^{t}=0.748$) and clearly performs better than it does in a generalist team ($s_{1}^{t}=0.7$). However, a specialist star in a baseline team that is heavily specialized in the “wrong” play type 3 ($s_{1}^{t}=0.588$) performs a lot worse than it does in a generalist team ($s_{1}^{t}=0.7$). Thus, while the performance effect of the specialist star performer differs among the various baseline teams, we cannot argue that he overall performs better or worse in generalist teams ($s_{1}^{t}=0.7$) than in specialist teams that are more to the left or to the right.

**Fig 2:**
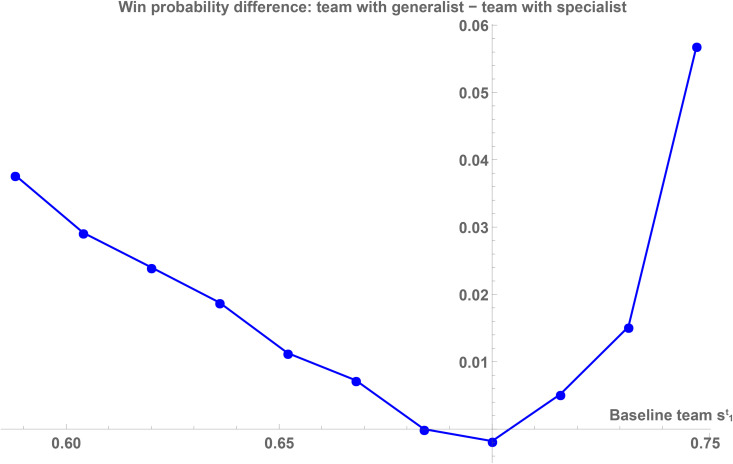
Comparison of the absolute performance of generalist star performers and specialist star performers for different baseline teams. For any type of baseline team, a team with a generalist is expected to outperform a team with a specialist. That is, for teams with specialist star performers, the negative performance effect of the increased predictability dominates the positive effect of using a superior play type. Illustrated is the absolute difference between the average win percentage of the different baseline teams with a generalist star performer and the average win percentage of the same teams with specialist star performers. A description of the variables used in the theoretical model is provided in S1 Table.

*Comparing a generalist to a specialist star performer has two opposing effects on team performance:* For a given defensive strategy, a specialist allows the team to focus on a particularly productive play type, increasing team performance. However, this also makes the strategy more *predictable*, allowing for more efficient defensive strategies which reduces team performance. While the net effect is ambiguous, we can use our simulation to explicitly calculate the net outcome. We compare the performance of a team with a generalist star with the performance of the same team with a specialist star for each of the 11 baseline teams. We consider the heterogeneity among specialists by taking averages over a specialist with a focus on play type 1 (*s*_1_ = 0.9695, $s_{2}=s_{3}=0.7$) and one with a focus on play type 3 (*s*_3_ = 0.9695, $s_{1}=s_{2}=0.7$). Fig 2 illustrates the outcome of this assessment. For any type of baseline team, a team with a generalist is expected to outperform a team with a specialist. That is, for teams with specialist star performers, *the negative performance effect of the increased predictability dominates the positive effect of using a specialized play type*. This gives rise to the following hypothesis:

#### Hypothesis 1 (H1).

Teams with generalist star performers outperform teams with specialist star performers.

**Fig 3:**
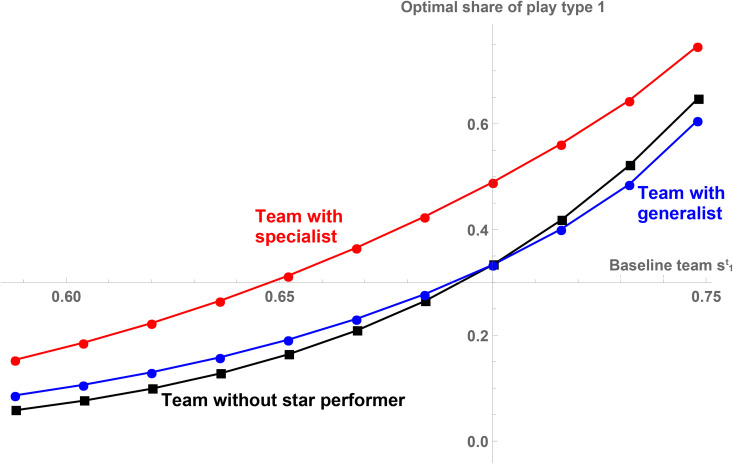
The share of play type 1 for the baseline teams with different star performers. Illustrated is the share of play type 1 for the different baseline teams without star performer (black squares), with a generalist star performer (blue circles), and with a specialist star performer (red circles). A description of the variables used in the theoretical model is provided in S1 Table.

Fig 3 shows explicitly how the teams make the best use of the skills of their baseline teams and, if present, their star performer by illustrating the (empirically observable) share of play type 1. First, consider teams without a star performer (black squares): The higher the team’s skill in play type 1 is, the more weight this team should put on this particular play type. However, the effect of the presence of a star performer on the optimal share of play type 1 depends on the type of the star performer: If player 1 is a generalist (blue circles), it is optimal to use a more balanced strategy (compared to the team without the star performer) which pushes the optimal share of play type 1, $\sigma_{1}$, towards 33.3%. In contrast, if the star performer is a specialist (red circles), it is optimal to adjust to the specialist skill set of the star performer by increasing the weight of play type 1. The effects on play types 2 and 3 are analogous.

We now use the outcome of the teams’ optimization, that is, the performance-maximizing shares of the three play types, to introduce the *Diversification Index*, a measure frequently used in the literature dealing with specialization and diversification [7,9]. This measure captures the diversification of play types used by the star performer in a particular game.


$$\begin{array}{c} \text{Diversification}=1-\sqrt{\sum_{p=1}^{P}w_{p}^{2}}, \end{array}$$ (4)

where *w*_*p*_ denotes the weight (or share) of play type *p* for the player or team under consideration (an analytical derivation of the play type weight for a particular player *w*_*p*_ from the team’s strategy can be found in S2 File). The diversification index is equal to 1 minus the calculated Euclidean distance. The higher the value, the higher the diversity of the star performer’s play type portfolio in a given game, i.e., the higher his multitasking capacity and the lower his degree of specialization.

**Fig 4:**
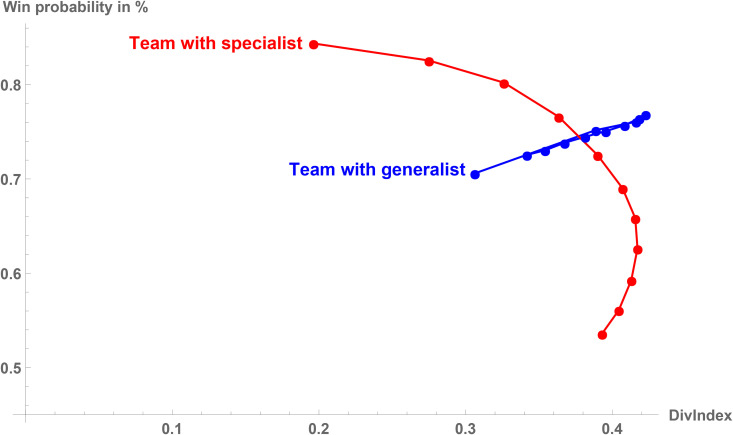
Star performers’ diversification and absolute team performance. Illustrated is the relationship between the diversification index of generalist and specialist star performers and absolute team performance. A description of the variables used in the theoretical model is provided in S1 Table. As robustness check, a relative performance measure is presented in S3 Fig.

Now consider the team performance in Fig 4. For the generalist, we observe a positive relation between the diversification index and the performance measure. That is, it is never beneficial for a generalist to adopt a less diversified play routine. For the specialist, it is actually possible that adopting a more diversified play style improves the team performance. This is particularly the case if baseline teams that are, without considering the star performer, already specialized in a particular play type (e.g., play type 1), use a star performer that is specialized in another play type (in our case always play type 3). The strong specialization of the baseline team on the “opposite” play type forces the team to focus strongly on this play type that does not benefit from the star performer. A slightly improved fit, that is, a baseline team that is less focused on the “opposite” play type, allows the team with the specialist star performer to move away from the “opposite” play type toward the play type in which the star excels. That is, the team can use a more diversified strategy, improving performance: This is the increasing part of the specialist curve in Fig 4. If we follow the curve further updward, the baseline team aligns more and more with the specialization of the star performer, making it optimal to focus on this play type and reducing diversification again: This is the downward-sloping part of the specialist curve in the upper part of Fig 4.

Thus, generalists and specialists differ fundamentally in the correlation between diversification and performance. The generalist is perfectly balanced, so the best use of his skills would be in a team that allows for maximum diversification. If the generalist plays in an unbalanced team that forces the team to choose a more specialized (low diversification) play style, performance will always suffer. In contrast, the specialist is, by definition, highly skilled in one particular play type (here, play type 1) but only average in the others. Whether higher diversification (i.e., a more balanced play style) correlates positively or negatively with team performance depends on “which side” of the specialized play style the team is coming from: If higher diversification means that the team focuses less on the strong play type of the specialist star performer, this hurts team performance. If, however, higher diversification means that the team moves away from focusing on the “wrong” play type (here, play type 3), team performance benefits. In other words, specialists are “asymmetric”, so whether moving toward a more generalized play style improves performance depends on whether the team is moving away from a specialization with a good or a bad fit between the specialist star performer and the team.

Note that teams always behave optimally in this model **–** there is no bias and no inefficiency. The (optimal) adjustment of the routine is triggered by a modified composition of the baseline team. We summarize our findings in the following hypotheses:

#### Hypothesis 2a (H2a).

If a generalist star performer chooses a more specialized play style, i.e., his diversification index reduces, team performance *always* declines.

#### Hypothesis 2b (H2b).

If a specialist star performer chooses a more generalized play style, i.e., his diversification index increases, team performance *may* improve.

## Empirical test

We test the hypotheses of the theoretical model using detailed on-court data coming from NBA games during four regular seasons (2012/13–2015/16). A unique feature of our data is comprehensive information about the play types that delineate the final outcome of all possessions for each team and player in each game during the study period (i.e., 104,330 player-game-level observations). This information is gathered by experienced video loggers for Synergy Sports Technology, a company that creates databases and analytics used by NBA coaching staffs and front offices. Overall, we have access to information for 4,913 games or 9,826 team-game-level observations. Due to missing information in key variables we had to exclude 306 overtime games. Moreover, we had to exclude 970 team-game-level observations characterized by the absence of a star performer. In general, an NBA player can miss a game due to injury, suspension, a coach’s decision (e.g., unprofessional behavior, rest) as well as personal reasons (e.g., the birth of a child). Since we focus on the impact of a star’s specialization on team performance, games in which a star was absent are naturally eliminated from our sample. Therefore, the final net estimation sample covers 8,244 team-game-level observations.

### Measures

*Performance measure.* In contrast to business firms where different stakeholders expect different outcomes from an organization, sports teams have a straightforward measure of success, i.e., win-loss records [27]. Given the focus of our analysis and the team-game-level structure of our data we use an absolute measure of team performance denoting the final outcome of a game (0 = loss, 1 = win).

*Star performers.* Following Kehoe et al. [5] we consider as star performers individuals demonstrating exceptional task performance in their teams, regardless of whether they enjoy star status outside their teams or not, i.e., so-called ‘performance stars’. In other words, we define stars relative to their teammates, rather than in comparison to the entire league, based on the premise that even in highly homogeneous teams, individual performance may never be entirely identical. Consequently, a star performer, according to our definition, can emerge in every team. To identify them, we utilize a measure frequently employed in the literature to determine talent [28], ability [29], and high performers [30] in NBA teams, i.e., the Estimated Wins Added (EWA). This measure captures a player’s overall contribution to his team, as it gives the estimated number of wins a player adds to the team’s season total above what a replacement player would produce [31]. Other measures frequently used are the Value Added (VA) [32] and the Player Efficiency Rating (PER) [33]. VA takes into account the number of points a player adds to his team’s season above what a replacement player would produce. PER is an estimate of a player’s per-minute productivity. Both metrics strongly correlate with EWA, resulting in a similar set of stars. Other studies have used All-Star status to identify star performers (e.g., [34]); this measure, however, also entails other attributes (e.g., players’ visibility and popularity) that go beyond pure performance. In this study, we focus on ‘performance stars’ as defined by Kehoe et al. [5]. Finally, several studies (e.g., [8]), utilize the Wins Produced (WP) measure. WP captures players’ contributions to their teams’ success [35]. While this metric also correlates with EWA, some differences arise due to the fact that WP tends to overvalue rebounding and thus favors players playing center/forward.

We define the player with the highest EWA among their teammates as the performance star. If a player with the highest EWA in a team has switched teams during the season, we consider as the star the player with the next-highest EWA who did not switch teams. Moreover, in case that two players share the highest EWA in their teams, we define as the star the player who has played comparatively more games. The average EWA of the star performers during the period of analysis was about 13.5, i.e., almost six times higher than the average league-wide EWA (2.4). The highest EWA values by season were achieved by LeBron James (Miami Heat, 2012/13), Kevin Durant (Oklahoma City Thunder, 2013/14) James Harden (Houston Rockets, 2014/15) and Stephen Curry (Golden State Warriors, 2015/16) respectively (see S3 Table).

*Generalist and specialist stars.* Previous research utilizing basketball data has focused on shot choices of guards (three-point shots) to define specialists under the premise that three-point shooting is a skill concentrated in a few and that most teams and players focus on two-point shots (e.g., [11]). Nowadays, however, three-point shooting is a skill that several NBA players possess (not only guards but also centers, e.g., Brook Lopez) and is the most common feature of scoring. It is indicative that in the last decade (2011–2021) the percentage of three-point shots went from 22% to 39%, while mid-range shots dropped from 31% to 13% [36]. Research utilizing soccer data (e.g., [37]) and football data (e.g., [38]) to measure specialization has focused on the tactical positions of players. In contrast to these sports, however, tactical positions in the NBA are less relevant, as these days players (and in particular stars) switch from guard to center depending on game conditions (see [39]). It is worth noting that across sports, athletes self-select into tactical positions based on physical capabilities and genetic characteristics (see [40]). Moreover, even within each tactical position, an athlete can undertake a variety of roles (e.g., in soccer a striker can play as a poacher, pressing forward, target man, false nine etc.; in football a running back can be a halfback, a fullback, a scat back, a power back etc.). The unique feature of our basketball data is that play types can be directly interpreted as task-specific human capital. We utilize the five most frequent offensive play types *p* at the player level, i.e., Pick & Roll Ball Handler, Isolation, Post Up, Spot Up, and Transition. According to our sample, on average, a star performer is involved in one of these five play types in about 8 out of 10 possessions per game while other offensive play types are less frequent. Based on this information, we compute our empirical index of Diversification_*i*,*g*_ that captures diversification of play types used by star performer *i* at game *g* along the lines of equation (4) in the theoretical model:


$$\begin{array}{c} {\text{Diversification}}_{i,g}=1-\sqrt{\sum_{p=1}^{5}{\left(\frac{{\text{Playtype}}_{p,i,g}}{{\text{Playtype}}_{i,g}}\right)}^{2}} \end{array}$$ (5)

We define as a generalist a star performer *i* at game *g* who falls within the top 33% of the Diversification_*i*,*g*_ distribution. Based on this distinction, we test H1. Note that our results are robust to other cut-offs, as well as when simply utilizing the continuous Diversification_*i*,*g*_ metric (see robustness checks in the Results section).

*Generalist and specialist teams.* Following our theoretical model, a generalist star is slightly more beneficial for generalist teams than for more specialized teams. At the same time, however, the quantitative effect of the “fit” between a generalist star and the different baseline teams seems to be negligible according to our simulation results. To empirically test the relevance of specialization/diversification of the star performer’s teammates, we identify generalist teams (top 33%) by calculating the degree of play type diversification of a team at the level of a game, explicitly *excluding* the star’s play type distribution. We then interact this variable with the variable denoting generalist stars. If the assumption made in the theoretical model holds, we should not find any significant moderating effect. Note that explicitly excluding the star keeps the team- and star-level diversification measures conceptually distinct. This is also reflected in Table 3, where the pairwise correlation between the two indices is low (i.e., r = 0.091).

**Table 3:** Descriptive statistics and correlation matrix.

No.	Variables	Mean	SD	Min	Max	1	2	3	4	5	6	7	8	9
1	Absolute performance	0.520	0.500	0	1	1								
2	Relative performance	1.016	0.144	0.569	1.757	0.8176	1							
3	Star diversification	0.406	0.096	0	0.553	0.0517	0.069	1						
4	Generalist star	0.333	0.471	0	1	0.0427	0.056	0.638	1					
5	Team diversification	0.485	0.038	0.263	0.552	0.0257	0.038	0.091	0.091	1				
6	Generalist team	0.334	0.472	0	1	0.0214	0.026	0.054	0.055	0.684	1			
7	Role switching	0.291	0.454	0	1	0.0321	0.037	0.249	0.225	0.061	0.051	1		
8	Average team salary (in million)	4.537	1.357	0.643	10.9	0.1382	0.133	0.017	0.031	0.057	0.071	0.006	1	
9	Home game	0.506	0.500	0	1	0.1841	0.204	0.006	0.025	0.018	0.004	−0.003	0.008	1

8,244 observations. All variables are measured at the game level. Absolute performance is measured by the win-loss dummy. Relative performance is measured by the relative point differential (points scored/points allowed). Results on relative performance and the metric specification of star and team diversification are provided in S4–S6 Tables.

**Fig 5:**
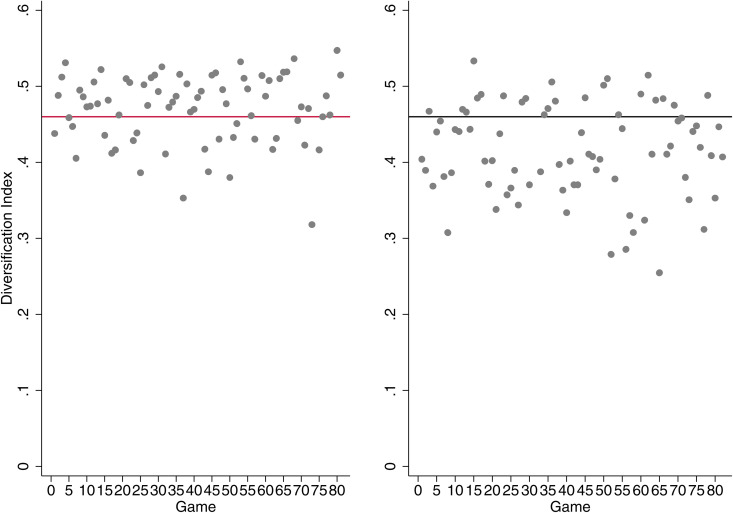
Diversification index at the game level of a generalist (left) and specialist (right) star at the season level. Overtime games and games in which the stars were absent are excluded. Games above the black line fall within the top 33% of the diversification distribution, i.e., the star performed as a generalist. LeBron James (left) acted as a generalist in 50 out of 76 games in the season 2015/16, while Stephen Curry (right) acted as a specialist in 56 out of 79 games in the season 2015/16.

*Role switching.* We include a variable (*role switching*) which takes into account the switching between roles, that is, whether a generalist (specialist) star at the season level performs as a specialist (generalist) at the game level. To do so, we identify generalist and specialist stars at the season level by calculating the overall degree of diversification of star performer *i* at season *t* and denote as generalists those who fall within the top 33% of the Diversification_*i*,*t*_ distribution. Fig 5 provides two indicative examples of a generalist (LeBron James) and a specialist (Stephen Curry) star performer at season level (season 2016) and their corresponding degree of diversification at game level. To test H2a and H2b, we interact *role switching* with the variable denoting generalist stars.

### Empirical strategy

In order to identify the effects of interest, we control for two variables frequently associated with team performance. *Average team salaries* are used to capture the impact of overall team quality (e.g., [41]). Since the variable is estimated at the game level, it also controls for game-by-game changes in quality due to absences (e.g., injuries or rest) and the departure or arrival of new talent (e.g., trades during the season). Moreover, since we expect home teams to outperform away teams, among others, due to referee bias [42], we control for *home games*. Finally, to control for unobserved heterogeneity across teams and seasons we include team (ψ) and season (θ) fixed effects. Likewise, we include opponent fixed effects (η) to control for opponent quality. Table 3 presents the means, standard deviations and the correlation matrix of all the variables used in our analysis.

The effects on the team’s *y* performance in game *g* in season *t* are analyzed in a regression framework of the following form:


$$\begin{array}{cc} {Performance}_{y,g,t}=\; & \alpha_{0}+\beta_{1}{GeneralistStar}_{y,g,t}\\ & +\beta_{2}{RoleSwitching}_{y,g,t}\\ & +\beta_{3}{GeneralistStar\times RoleSwitching}_{y,g,t}\\ & +\beta_{4}{ControlVariables}_{y,g,t}\\ & +\psi_{y}+\eta_{g}+\theta_{t}+\epsilon_{y,g,t} \end{array}$$ (6)

Since our models include higher-order terms, the variables are standardized. As fixed effects conditional maximum likelihood estimators suffer from the incidental parameters problem (see [43]) and the estimation of mean marginal effects can be particularly challenging in nonlinear models with fixed effects (see [44]), we implement fixed effects linear probability models (LPM) to test our hypotheses. Since the estimations include two observations for each game, we implement robust standard errors clustered at the game level. We also use clustering at the team level as well as at the dyad level, i.e., for games between the same pair of teams. Overall, our main findings remain (results are available upon request).

## Results

Table 4 provides the parameter estimates that can be directly interpreted as the mean marginal effects of covariates on the absolute team performance (i.e., win-loss). We first regress the performance measure on a set of control variables and then we include our variables of interest stepwise.

**Table 4:** Regression coefficients on absolute performance.

Variables	(1)	(2)	(3)	(4)	(5)	(6)
Generalist star		0.031***	0.031***	0.029**	0.027*	0.099***
		(0.011)	(0.011)	(0.011)	(0.014)	(0.018)
Generalist team			0.004	0.003	0.002	0.004
			(0.011)	(0.011)	(0.014)	(0.011)
Role switching				0.012	0.012	0.086***
				(0.012)	(0.012)	(0.019)
Generalist star x Generalist team					0.004	
					(0.022)	
Generalist star x Role switching						−0.164***
						(0.032)
Average team salary	0.039***	0.038***	0.038***	0.038***	0.038***	0.034***
	(0.006)	(0.006)	(0.006)	(0.006)	(0.006)	(0.006)
Home game	0.179***	0.178***	0.178***	0.178***	0.179***	0.178***
	(0.014)	(0.014)	(0.014)	(0.014)	(0.014)	(0.014)
Team FEs	yes	yes	yes	yes	yes	yes
Opponent FEs	yes	yes	yes	yes	yes	yes
Season FEs	yes	yes	yes	yes	yes	yes
Constant	0.424***	0.408***	0.407***	0.402***	0.403***	0.328***
	(0.015)	(0.016)	(0.016)	(0.016)	(0.017)	(0.022)
R-squared	0.165	0.166	0.166	0.166	0.166	0.169

8,244 observations. Absolute performance is measured by the win-loss dummy. Robust clustered standard errors by game (4,552 clusters) in parentheses. Significance levels are indicated as *** p < 0.01, ** p < 0.05, * p < 0.1.

Overall, and regardless of the specification, we find that both average team salary and home games are positively related to the probability of winning, with the size and significance of the controls remaining stable. Moreover, consistent with our theoretical model, generalist stars exert a positive effect on performance (H1). In particular, we find that teams in which the star functions as generalist in a given game have an about 3 percentage points higher probability of winning. Interestingly, the indicator variable that accounts for whether the star’s teammates function as generalists is positive but not statistically significant. Likewise, the two-way interaction term between generalist teams and generalist stars is positive but not statistically significant. Both findings are in line with our theoretical model as well as the corresponding simulation results.

Furthermore, while role switching *per se* does not entail any statistically significant effect, we find that the type of switching matters. More precisely, supporting the theoretical model and the numerical simulations, we find that team performance in a given game deteriorates when generalist stars (at the season level) switch to specialist stars (at the game level) (H2a), while team performance in a game improves when specialist stars (at the season level) switch to generalist stars (at the game level) (H2b). Table 5 provides the predicted values for all four possible alternatives, that is, teams with (i) a specialist star at the season level who remained a specialist at the game level (no switch), (ii) a specialist star at the season level who switched to a generalist at the game level, (iii) a generalist star at the season level who remained a generalist at the game level (no switch), (iv) a generalist star at the season level who switched to a specialist at the game level. In detail, it shows that when no switching takes place, teams with a generalist star outperform teams with a specialist star by about 2 percentage points in terms of win probability. When a specialist star switches to a generalist in a particular game, the win probability increases by about 9 percentage points compared to no switch. For a generalist star, switching to a specialist decreases the win probability by about 8 percentage points compared to no switch. All in all, it becomes apparent that teams perform better with generalist stars than with specialist stars.

**Table 5:** The predictive margins based on the role switching.

Role switching	Absolute performance
Specialist star (season) → Specialist star (game)	0.486***
	(0.006)
Specialist star (season) → Generalist star (game)	0.572***
	(0.015)
Generalist star (season) → Generalist star (game)	0.585***
	(0.013)
Generalist star (season) → Specialist star (game)	0.507**
	(0.013)

Average marginal effects based on the interaction effects reported in Model 6 of Table 4. Absolute performance is measured by the win-loss dummy. Robust standard errors by game in parentheses. Significance levels are indicated as *** p < 0.01, ** p < 0.05, * p < 0.1.

In order to test the robustness of these findings, we re-estimate our models with different specifications and samples. *First*, similar to the numerical simulations, we use an alternative measure which aims to capture the relative team performance. This variable is measured as the natural logarithm of the relative point differential (points scored/points allowed). The regression coefficients and the predictive margins are provided in S4 and S5 Tables, respectively. Our main findings remain. *Second*, instead of the binary specification denoting generalist teams and stars, we use the metric specification of the diversification variables. This specification avoids setting any arbitrary thresholds, preserves variance, and reduces information loss. Results of these models are provided in S6 Table and show that team performance benefits from higher star diversification. Moreover, these results confirm that the effect of role switching on performance depends on the degree of diversification. As a further robustness check, we also estimated models splitting Diversification_*i*,*g*_ into quartiles (top 25%) and quantiles (top 20%). Our main findings remain (results are available upon request). *Third*, since role switching is a choice, endogeneity concerns may arise. However, by re-estimating our models excluding all games in which role switching took place, we still find that teams with generalist stars outperform teams with specialist stars (see S7 Table). Moreover, there might be a potential selection bias inherent in good teams actively seeking and selecting individuals who possess diverse skill sets. This selection bias could impact the findings related to the performance advantages attributed to generalist stars. While we could address this issue in our theoretical model, where all teams perform equally when the star is excluded, addressing this issue empirically is challenging. Each team’s unique situation (e.g., gaps in skills or positions), varying recruitment strategies, the qualitative nature of the selection process (e.g., scouting reports, head managers’ and coaching staff opinions, etc.), as well as a wide range of intangible factors (e.g., a player’s willingness to join a team, anticipated player-team chemistry, etc.), mean that any empirical approach might not fully capture the complexity of this selection bias. *Fourth*, in our theoretical model, we are able to calibrate the generalist and the specialist star such that in a 1-vs-1 game between them, each wins with a probability of 50%. In our empirical model, it is difficult to compare the fundamental abilities or talents that a player possesses inherently, independent of external factors such as team dynamics, coaching, or overall team performance. The lack of a metric that can assess the skills a player naturally brings to the game prevents us from empirically determining whether generalists and specialists have comparable “quality”. As an indirect test, we examined whether team performance is affected differently when a specialist or generalist star performer (defined at the season level) is absent from a game. Absence is defined as injury-related unavailability. Our results indicate that, as expected, teams with generalist stars outperform teams with specialist stars, and the absence of a star performer (regardless of type) negatively affects team performance. However, there is no statistically significant difference in this effect based on whether the absent star is a generalist or a specialist. This indicates that the relative importance of both types of star performers to their teams is comparable (see S8 Table). *Finally*, both in our theoretical model and in our empirical estimations, we define one star performer per team. However, in real-world settings, teams may also employ several stars. Arguably, the impact of star diversification is likely to be moderated by the existence of multiple stars, as the degree of dependency on a star diminishes with the presence of additional stars (see [34,45]). To discriminate between one-star and multiple-star teams, we first estimate the average EWA at the team level and then the EWA difference between the players with the two highest EWA in each team. If the EWA difference between the top two players is greater (smaller) than the average EWA at the team level, we consider these teams to be one-star (multiple-star) teams (see S9 Table). We run estimations on a sub-sample consisting only of one-star teams and on a sub-sample consisting only of multiple-star teams (see S10 Table). Our main findings remain when we consider one-star teams, with the sizes of the coefficients for the variables accounting for star diversification and role switching increasing substantially. In contrast, star diversification and role switching seem to have no effect on multiple-star teams’ performance. Therefore, we conclude that our findings hold only for teams that rely on a unique star. To further explore how and why results differ between one-star and multi-star teams, and whether star dependence is the mechanism, we examine effect differences between scenarios in which a generalist or specialist star (defined at the season level) is injured in either one- or multi-star teams. This additional robustness check points out: In one-star teams, the impact of a star’s absence depends on whether the star is a generalist or a specialist. The findings indicate that losing a generalist is far more detrimental than losing a specialist. By contrast, in multi-star teams we cannot confidently conclude that the absence effect differs by star type. In other words, these subsample estimations suggest that one-star teams are more dependent on generalist stars than multi-star teams S8 Table.

## Discussion and conclusion

Star performers are widely regarded as crucial for organizational success, yet little is known about how their degree of specialization or diversification affects team performance. By linking research on star employees with the literature on specialization and diversification, we addressed this gap, focusing on non-executive employees in team environments within a basketball setting. Introducing an adaptive role framework, we considered the possibility that star performers could switch roles between specialization and diversification, depending on situational demands.

Using a game-theoretical model based on the O-Ring production function and solving the model by the Nash equilibrium in mixed strategies, we illustrated how the skills of individual players determine aggregate team skills and how team skills in turn determine performance. Specialization has two opposing effects: It allows a team to focus on few play types in which it truly excels, but it also makes the team more predictable for the opposing defense. By varying the team composition and the type of star, we used numerical simulations of the theoretical model to infer their *causal* impact on performance. The model revealed that teams with generalist stars tend to outperform those with specialist stars. Moreover, the impact of role-switching varies by star type: while generalist stars switching to specialist roles reduce team performance, specialist stars transitioning to generalist roles may enhance performance. Our empirical analysis using NBA data supported these predictions, demonstrating that generalist stars contribute more to team success and that role-switching dynamics align with theoretical expectations.

Utilizing a sports setting allowed us to leverage detailed, individual-level data to identify star performers and assess their specialization or diversification—a task that is often difficult in non-sports organizations. While this makes sports an invaluable context for testing organizational theories, our findings—rooted in the specific dynamics of basketball—come with important boundary conditions. Because sports can differ markedly from other industries, and sports themselves vary in task structure, it is crucial to consider how these contextual differences may shape conclusions.

When considering generalizability across sports, basketball’s own structure presents another contingency. Its reciprocal interdependence contrasts with other team sports. As prior work on leadership and task interdependence suggests, moderating effects can arise (see [46]). Baseball exemplifies pooled interdependence, while football and soccer are closer to sequential interdependence. The strategic advantage of a generalist star that reduces predictability in basketball may be less salient in more structured or modular contexts. Moreover, basketball’s smaller team size amplifies a star’s influence. In sports with larger squads, such as football and soccer, an individual’s impact (even that of a star) is likely to be more diluted (see [47]). Notwithstanding, developing reliable measures of diversification/specialization in soccer or football, at the player level and across positions, remains challenging. For instance, Simmons and Berri [38] use football data to exploit the distinct tasks of running backs to identify specialization, but running backs are less likely than quarterbacks or wide receivers to be star performers at the team or league level.

Regarding generalizability beyond sports, and following prior empirical work using NBA data (see [29,48]), our findings may apply to settings where (action) teams rely on a unique star, are characterized by cross-functional and reciprocal interdependence, and operate in highly visible, competitive environments (e.g., certain software development teams). However, the extent of interdependence varies across industries. We therefore expect our conclusions to be moderated by organizational features. For example, in environments with pooled interdependence (e.g., largely independent contributions in an R&D lab) or sequential interdependence (e.g., a manufacturing assembly line), deep specialization may be more valuable, dampening the benefit of a generalist star performer. This points to the importance of testing how differences in stars’ individual qualities affect performance under varying levels of task interdependence (see [49]). Organizational structure may also matter: in hierarchical organizations with rigid roles, a specialist star’s depth could be the primary source of value, whereas in flatter, collaborative structures (e.g., tech startups), a generalist star’s breadth may be more consequential.

In summary, the optimal degree of a star performer’s specialization may be highly contingent on context. Recognizing these boundary conditions underscores the need for future research across different types of teams and industries to examine how task interdependence, team size, and organizational structure moderate the relationship between a star’s diversification and team performance. Such work would clarify when star diversification is most beneficial and inform organizational design and talent-management strategies.

Turning to limitations, *first*, our empirical analysis examines whether observed patterns align with the theoretical predictions of our game-theoretical model, focusing on identifying correlations rather than establishing causal relationships. As such the empirical part should be treated as correlational assessment of the formal theoretical model rather than a causal exercise. Note, however, that our goal is to provide initial evidence and directions for future research. *Second*, both the theoretical and empirical analyses assume that stars/teams seek to maximize scoring probabilities irrespective of game state. While we argue that winning or losing does not directly alter play type composition, score dynamics may affect effort provision (e.g., [50]), indirectly influencing play selection. Future work could examine how game contexts shape play type composition. *Third*, given the difficulty of measuring defensive ability (e.g., [51]), we focus on a single, transparent, precisely measurable, and arguably very important task in our chosen setting for constructing our diversification measure, i.e., offensive play types. We note, however, that generalist stars may also create value through defensive versatility (e.g., switching across positions), a channel not captured by our data but consistent with our mechanism. Developing a broader, multidimensional construct that can capture the overall degree of star diversification (offense, defense, shooting styles, positional fluidity, etc.) remains an important task for future research. *Fourth*, although the NBA (and basketball more broadly) has continued to evolve (e.g., “positionless” lineups and further growth in three-point rates), our theoretical predictions are not era-specific, and our window (2012–2016) captures the early phase of these strategic shifts. While we see no *a priori* reason to expect systematic changes in our findings, the increased spacing and switching of the modern game plausibly amplify the value of star diversification; nonetheless, it would be valuable to test whether the influence of star diversification has increased or declined over time. *Fifth*, in multi-star settings lineup-level complementarity may arise: multiple specialist stars could collectively approximate a “functional generalist”, distributing play types such that predictability falls without any single star diversifying. While this mechanism is theoretically plausible, our design is not powered to isolate it, so we treat it as a boundary condition of our findings. Future work could model lineup-level diversification and exploit play-by-play lineup information and substitutions to map complementarities over possessions. Despite these limitations, our theoretical model offers a first formal framework for studying how differences in individual star qualities relate to team performance. While the provided empirical evidence from the NBA is theory-consistent, we hope that this study paves the way for future (empirical) work in other sports and beyond.

## Supporting information

S1 Fig Robustness check: star performers’ diversification and team performance with alternative definition of specialist star performer. This figure illustrates how the absolute team performance (% of games won) with a generalist or a specialist is affected by the diversification of the teams’ play routine. In this robustness check, we keep our baseline teams (without the star) and the generalist star unchanged, but the specialist is now specialized in play type 3 instead of play type 1. The results remain (qualitatively) unchanged and replicate the findings from Fig 4: For generalists, we observe a positive relation between the diversification index and team performance. It is never beneficial for a generalist to adopt a less diversified play routine. For teams with a specialist, a more diversified play style can improve team performance if baseline teams are already specialized in a particular play type but use a star performer who is specialized in another play type. A slightly improved fit, that is, a baseline team that is less focused on the “opposite” play type, allows the team with the specialist to use a more diversified strategy, improving performance: This is the increasing part of the specialist curve. If we follow the curve further upward, the baseline team aligns more and more with the specialization of the star performer, making it optimal to diversification again: This is the downward-sloping part of the specialist curve in the upper part.
(PDF)

S2 Fig Robustness check: relative team performance (points scored/points allowed) without star performer vs. with generalist vs. with specialist. While Fig 1 focuses on absolute performance, this diagram provides a robustness check and illustrates the simulated relative performance of the 11 different baseline teams without a star performer (black squares), with a generalist star performer (blue circles) and with a specialist star performer (red circles). Relative performance takes into account the relative point differential (points scored/points allowed). Without a star performer (black squares), there is no correlation between the different baseline teams and their performance. For teams in which player 1 is a star performer, performance benefits significantly from both types of stars. While a generalist star is slightly more beneficial for generalist teams (in the middle) than for more specialized teams (that is, baseline teams more to the left or to the right), the quantitative effect of the generalist’s fit seems negligible. In contrast, a specialist star is best utilized in a baseline team that is heavily specialized toward the strength of the generalist in play type 1 (right) and clearly performs better than he does in a generalist team (middle) or in a team that is specialized in the “wrong” play type 3 (left).
(PDF)

S3 Fig Robustness check: star performers’ diversification and relative team performance. While Fig 4 focuses on absolute performance, this diagram provides a robustness check and illustrates how the relative team performance (points scored/points allowed) with a generalist or a specialist is affected by the diversification of the teams’ play routine. For generalists, we observe a positive relation between the diversification index and team performance. It is never beneficial for a generalist to adopt a less diversified play routine. For teams with a specialist, a more diversified play style can improve the team performance if baseline teams are already specialized in a particular play type, but use a star performer that is specialized in another play type. A slightly improved fit, that is, a baseline team that is less focused on the “opposite” play type, allows the team with the specialist to use a more diversified strategy, improving performance: This is the increasing part of the specialist curve. If we follow the curve further upward, the baseline team aligns more and more with the specialization of the star performer, making it optimal to diversification again: This is the downward-sloping part of the specialist curve in the upper part.
(PDF)

S1 Table Variables of the theoretical model. These variables are used in the theoretical model “A simple (theoretical) game of basketball”.
(PDF)

S2 Table Definition of play types. (PDF)

S3 Table Overview of star performers by season and team. Data were retrieved from Hollinger NBA Player Statistics published on ESPN.com; the average league-wide Estimated Wins Added (EWA) for the season 2012/13, 2013/14, 2014/15, and 2015/16 is based on 531, 549, 576 and 529 NBA players respectively; * denotes a player with the second (or third) highest EWA in his team if the player with the highest EWA has switched teams during the season; ** denotes a player sharing the highest EWA with another player on the team, but has played more games. In italics are generalist stars at the season level, i.e., stars who fall within the top 33% of the distribution.
(PDF)

S4 Table Regression coefficients on relative performance. 8,244 Observations. Relative performance is measured by the natural logarithm of the relative point differential (points scored/points allowed). Robust clustered standard errors by game (4,552 clusters) in parentheses. Significance levels are indicated as *** p < 0.01, ** p < 0.05, * p < 0.1.
(PDF)

S5 Table The predictive margins based on the role switching. Average marginal effects based on the interaction effects reported in model 6 in S4 Table. Relative performance is measured by the natural logarithm of the relative point differential (points scored/points allowed). Robust standard errors by game in parentheses. Significance levels are indicated as *** p < 0.01, ** p < 0.05, * p < 0.1.
(PDF)

S6 Table Regression coefficients using the metric specification. 8,244 Observations. Absolute performance is measured by the win-loss dummy. Relative performance is measured by the natural logarithm of the relative point differential (points scored/points allowed). Robust clustered standard errors by game (4,552 clusters) in parentheses. Significance levels are indicated as *** p < 0.01, ** p < 0.05, * p < 0.1.
(PDF)

S7 Table Regression coefficients based on non-switchers subsample. 5,841 Observations. Absolute performance is measured by the win-loss dummy. Relative performance is measured by the natural logarithm of the relative point differential (points scored/points allowed). Robust clustered standard errors by game (3,977 clusters) in parentheses. Significance levels are indicated as *** p < 0.01, ** p < 0.05, * p < 0.1.
(PDF)

S8 Table Regression coefficients based on star absence and team composition (one-star vs. multiple-star teams). As dependent variable serves the absolute performance (win-loss dummy). To discriminate between one-star and multiple-star teams, we first estimate the average EWA at the team level and then the EWA difference between the two players with the highest EWA in each team. If the EWA difference between the top two players is greater (smaller) than the average EWA at the team level, we consider these teams to be one-star (multiple-star) teams. We code absence only for injuries; suspensions, rest management, and personal reasons are excluded. EWA: Estimated Wins Added. Robust clustered standard errors by game in parentheses. Significance levels are indicated as *** p < 0.01, ** p < 0.05, * p < 0.1.
(PDF)

S9 Table List of multiple-star teams by season. To identify multiple-star teams, we first estimate the average EWA at the team level and then the EWA difference between the two players with the highest EWA in each team. If the EWA difference between the top two players is greater (smaller) than the average EWA at the team level, we consider these teams to be one-star (multiple-star) teams. EWA: Estimated Wins Added.
(PDF)

S10 Table Regression coefficients based on team composition (one-star vs. multiple-star teams). As dependent variable serves the absolute performance (win-loss dummy). To discriminate between one-star and multiple-star teams, we first estimate the average EWA at the team level and then the EWA difference between the two players with the highest EWA in each team. If the EWA difference between the top two players is greater (smaller) than the average EWA at the team level, we consider these teams to be one-star (multiple-star) teams. EWA: Estimated Wins Added. Robust clustered standard errors by game in parentheses. Significance levels are indicated as *** p < 0.01, ** p < 0.05, * p < 0.1.
(PDF)

S1 File Definition of Nash Equilibrium. (PDF)

S2 File Derivation of *w*_*p*_. (PDF)
