# Supplementary figures and images for: Exploring the impact of specialist and generalist stars on organizational performance
Source: PLoS One. 2026 May 28;21(5):e0349682. doi: 10.1371/journal.pone.0349682 (PMC13218541; doi:10.1371/journal.pone.0349682)

Win probability in %

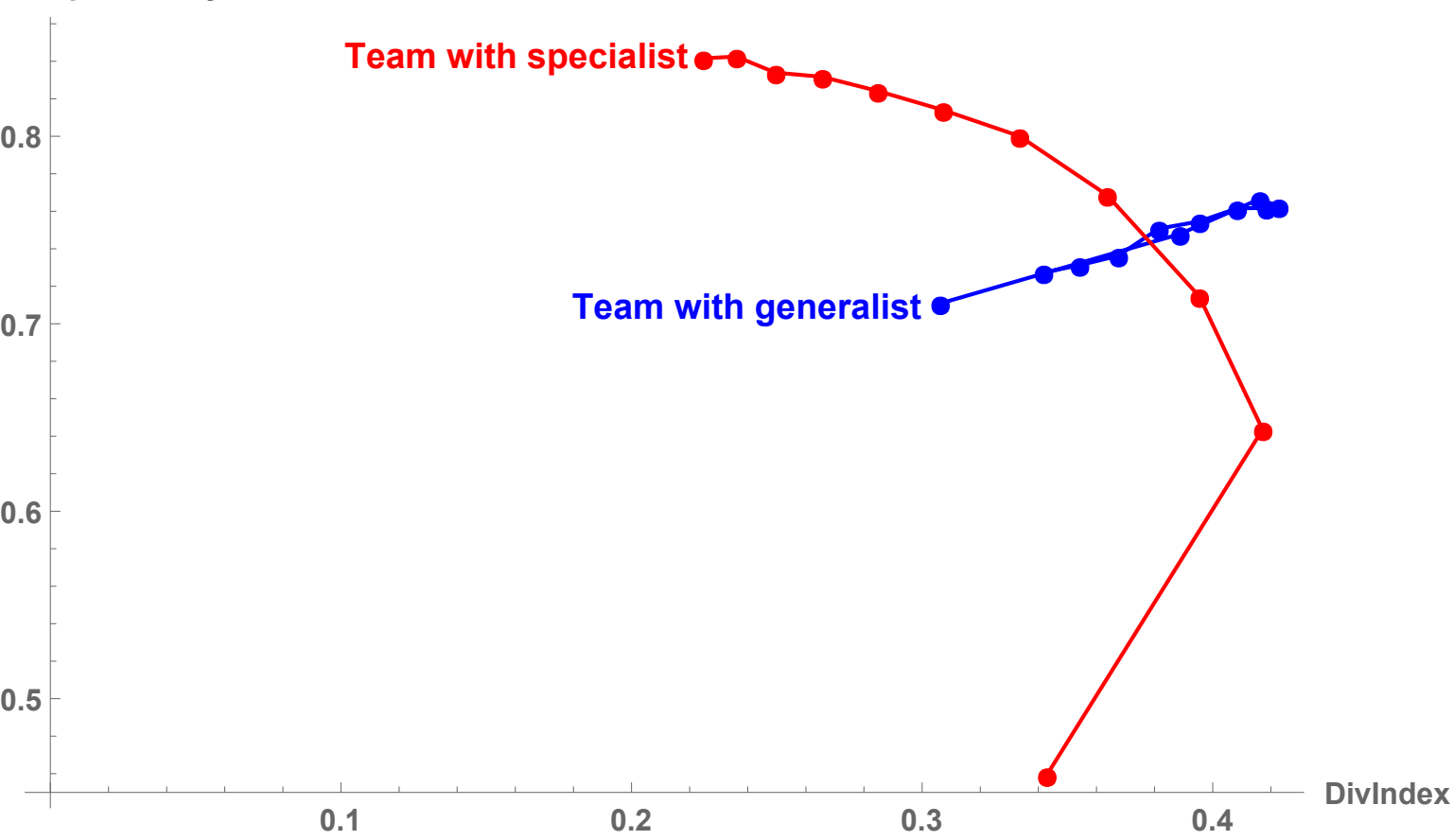

Supplement: S1 Fig — This figure illustrates how the absolute team performance (% of games won) with a generalist or a specialist is affected by the diversification of the teams’ play routine. In this robustness check, we keep our baseline teams (without the star) and the generalist star unchanged, but the specialist is now specialized in play type 3 instead of play type 1. The results remain (qualitatively) unchanged and replicate the findings from Fig 4: For generalists, we observe a positive relation between the diversification index and team performance. It is never beneficial for a generalist to adopt a less diversified play routine. For teams with a specialist, a more diversified play style can improve team performance if baseline teams are already specialized in a particular play type but use a star performer who is specialized in another play type. A slightly improved fit, that is, a baseline team that is less focused on the “opposite” play type, allows the team with the specialist to use a more diversified strategy, improving performance: This is the increasing part of the specialist curve. If we follow the curve further upward, the baseline team aligns more and more with the specialization of the star performer, making it optimal to diversification again: This is the downward-sloping part of the specialist curve in the upper part. (PDF) [file pone.0349682.s001.pdf]

Point difference in %

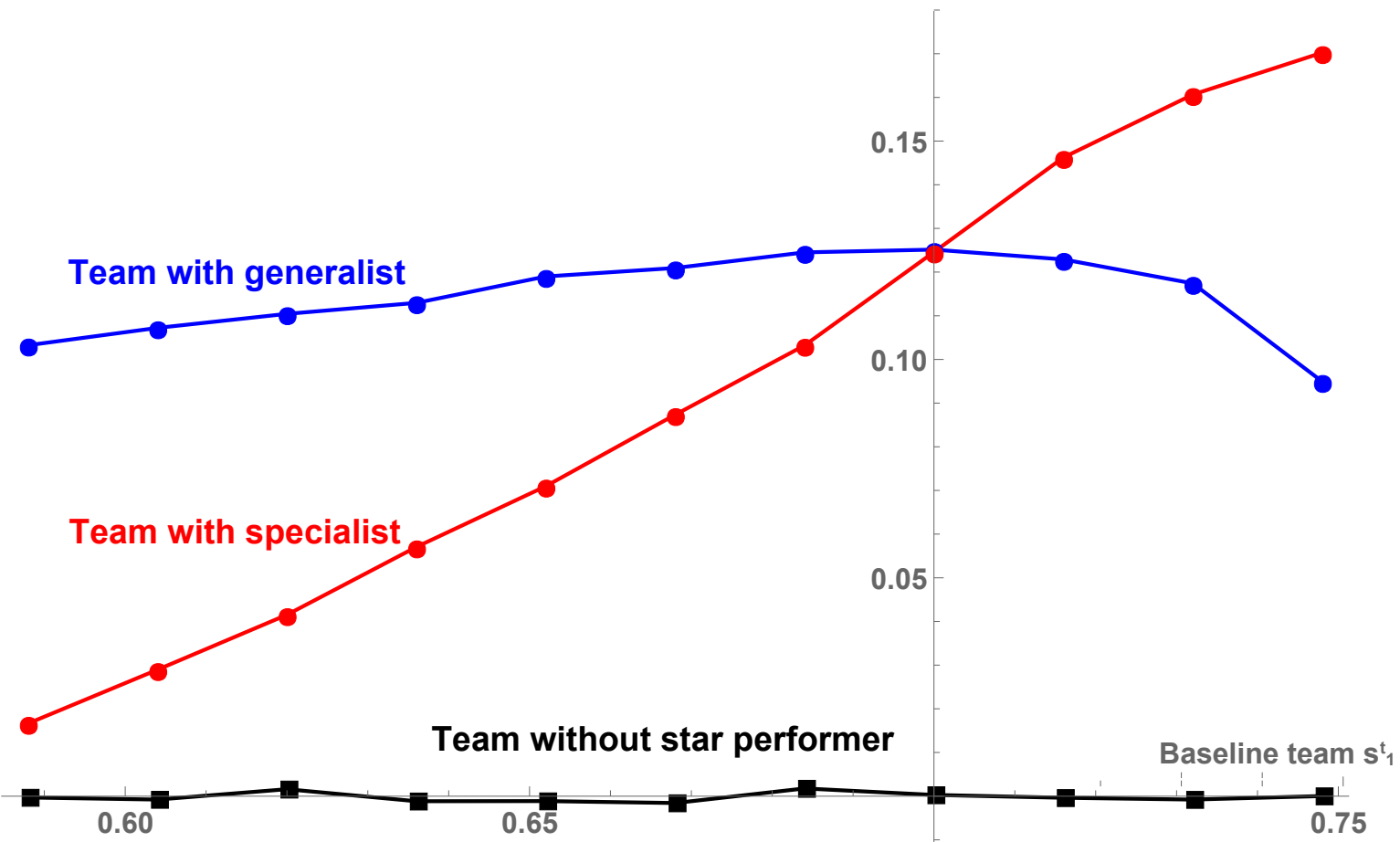

Supplement: S2 Fig — While Fig 1 focuses on absolute performance, this diagram provides a robustness check and illustrates the simulated relative performance of the 11 different baseline teams without a star performer (black squares), with a generalist star performer (blue circles) and with a specialist star performer (red circles). Relative performance takes into account the relative point differential (points scored/points allowed). Without a star performer (black squares), there is no correlation between the different baseline teams and their performance. For teams in which player 1 is a star performer, performance benefits significantly from both types of stars. While a generalist star is slightly more beneficial for generalist teams (in the middle) than for more specialized teams (that is, baseline teams more to the left or to the right), the quantitative effect of the generalist’s fit seems negligible. In contrast, a specialist star is best utilized in a baseline team that is heavily specialized toward the strength of the generalist in play type 1 (right) and clearly performs better than he does in a generalist team (middle) or in a team that is specialized in the “wrong” play type 3 (left). (PDF) [file pone.0349682.s002.pdf]
