# Supplementary material for: Exploring the impact of specialist and generalist stars on organizational performance
Source: PLoS One. 2026 May 28;21(5):e0349682. doi: 10.1371/journal.pone.0349682 (PMC13218541; doi:10.1371/journal.pone.0349682)
Supplement: S3 Fig — While Fig 4 focuses on absolute performance, this diagram provides a robustness check and illustrates how the relative team performance (points scored/points allowed) with a generalist or a specialist is affected by the diversification of the teams’ play routine. For generalists, we observe a positive relation between the diversification index and team performance. It is never beneficial for a generalist to adopt a less diversified play routine. For teams with a specialist, a more diversified play style can improve the team performance if baseline teams are already specialized in a particular play type, but use a star performer that is specialized in another play type. A slightly improved fit, that is, a baseline team that is less focused on the “opposite” play type, allows the team with the specialist to use a more diversified strategy, improving performance: This is the increasing part of the specialist curve. If we follow the curve further upward, the baseline team aligns more and more with the specialization of the star performer, making it optimal to diversification again: This is the downward-sloping part of the specialist curve in the upper part. (PDF) [file pone.0349682.s003.pdf]

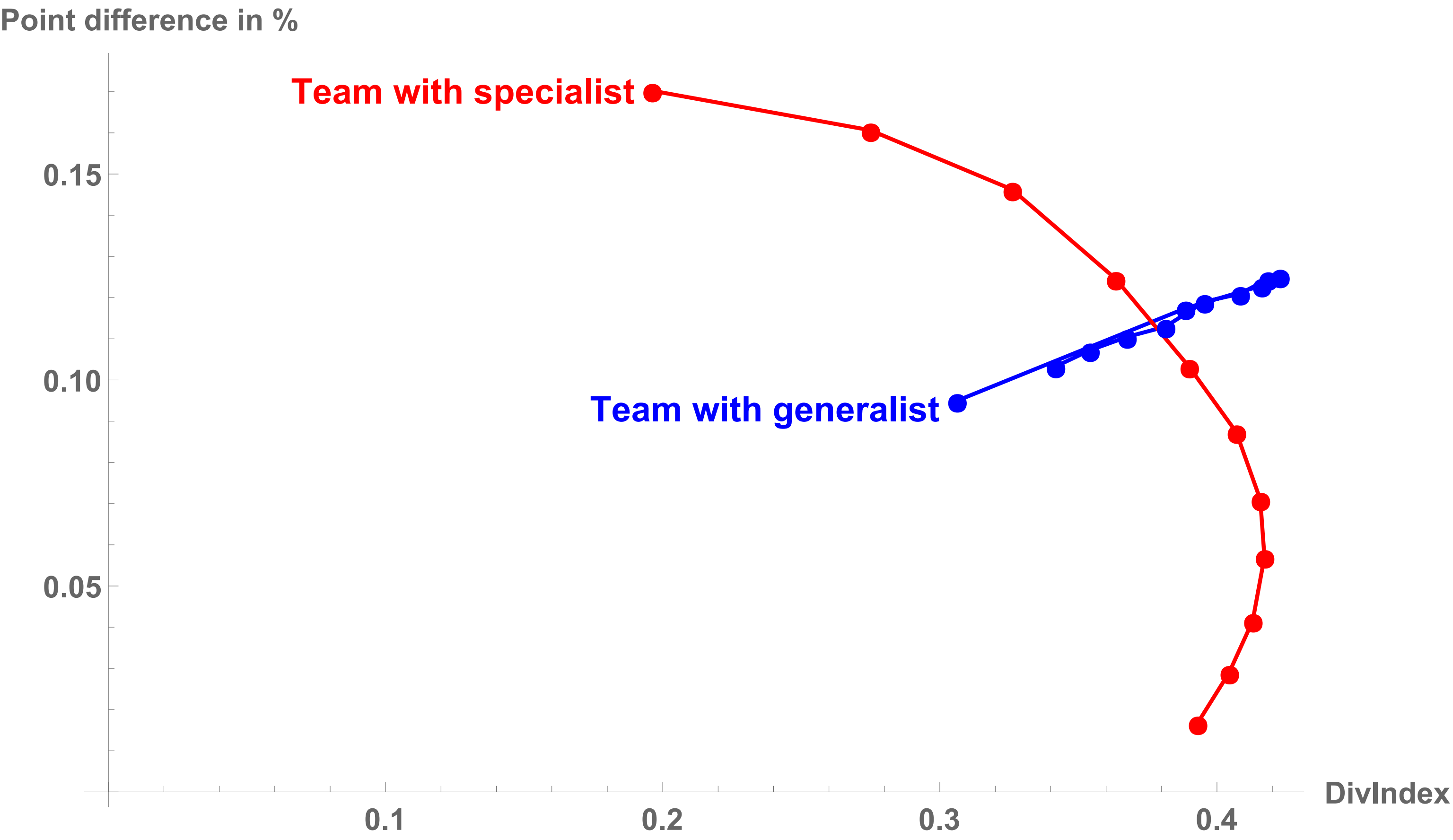

Point difference in %
Team with specialist
Team with generalist
0.15
0.10
0.05
0.1
0.2
0.3
0.4
DivIndex
