# Supplementary material for: Exploring the impact of specialist and generalist stars on organizational performance
Source: PLoS One. 2026 May 28;21(5):e0349682. doi: 10.1371/journal.pone.0349682 (PMC13218541; doi:10.1371/journal.pone.0349682)
Supplement: S1 Table — These variables are used in the theoretical model “A simple (theoretical) game of basketball”. (PDF) [file pone.0349682.s004.pdf]

Variable	Description
d_n	Individual defensive skill of player n
D	Team skill playing defense (aggregated)
δ_p	Weight that the defense puts on defending against play type p
$\delta = (\delta_1, \delta_2, ..., \delta_P)$	Strategy of the defense
$n \in \{1, 2, 3, 4, 5\}$	Index for player 1 to 5
P	Number of available different offensive play types
$p \in \{1, 2, ..., P\}$	Identifies a particular offensive play type
$\varphi(S_p, D, \delta_p)$	Scoring probability of play type p for given offensive and defensive skills and defensive focus δ_p
$s_{n,p}$	Individual skill in offensive play type p of player n
S_p	Team skill using offensive play type p (aggregated)
σ_p	Probability that the offense chooses play type p
$\sigma = (\sigma_1, \sigma_2, ..., \sigma_P)$	Strategy of the offense
t	Indicates an average team member
$\omega_{m,p}$	Probability that player m finishes play type p
$w_{m,p}$	Weight of play type p for player m
