# Supplementary material for: Exploring the impact of specialist and generalist stars on organizational performance
Source: PLoS One. 2026 May 28;21(5):e0349682. doi: 10.1371/journal.pone.0349682 (PMC13218541; doi:10.1371/journal.pone.0349682)
Supplement: S2 Table — (PDF) [file pone.0349682.s005.pdf]

Transition: When the possession-ending event comes before the defense sets following a possession change and a transition from one end of the court to the other.
Isolation: When the possession-ending event is created during a "one-on-one" matchup.
Pick & Roll – Ball Handler: A screen is set on the ball handler's defender out on the perimeter. The offensive player can use the screen or go away from it. As long as the play yields a possession-ending event, it is tagged as a pick-and-roll.
Pick & Roll – Roll Man: When a screen is set for the ball handler, and the screen setter then receives the ball for a possession-ending event.
Post-Up: When an offensive player receives the ball with his back to the basket and is less than 15' from the rim when the possession-ending event occurs.
Spot-Up: When the possession-ending event is a catch-and-shoot or catch-and-drive play.
Hand-Off: The screen setter starts with the ball and hands it to a player cutting nearby.
Cut: An interior play in which the finisher catches a pass while moving toward, parallel to or slightly away from the basket.
Off Screen: Identifies players coming off of screens going away from the basket toward the perimeter.
Rebound (Putbacks): When the rebounder attempts to score before passing the ball or establishing themselves in another play type.
Miscellaneous: When the action does not fit any of the other play types. This includes (amongst others) last-second full-court shots and fouls in the backcourt.
