# Supplementary material for: Exploring the impact of specialist and generalist stars on organizational performance
Source: PLoS One. 2026 May 28;21(5):e0349682. doi: 10.1371/journal.pone.0349682 (PMC13218541; doi:10.1371/journal.pone.0349682)
Supplement: S3 Table — Data were retrieved from Hollinger NBA Player Statistics published on ESPN.com; the average league-wide Estimated Wins Added (EWA) for the season 2012/13, 2013/14, 2014/15, and 2015/16 is based on 531, 549, 576 and 529 NBA players respectively; * denotes a player with the second (or third) highest EWA in his team if the player with the highest EWA has switched teams during the season; ** denotes a player sharing the highest EWA with another player on the team, but has played more games. In italics are generalist stars at the season level, i.e., stars who fall within the top 33% of the distribution. (PDF) [file pone.0349682.s006.pdf]

	Season 2012/13		Season 2013/14		Season 2014/15		Season 2015/16	
**Team**	**Star performer**	**EWA**	**Star performer**	**EWA**	**Star performer**	**EWA**	**Star performer**	**EWA**
ATL	*Al Horford*	12,7	*Paul Millsap*	10,3	*Al Horford*	12,5	*Paul Millsap*	13,0
BKN	Brook Lopez	15,9	Deron Williams	6,9	Brook Lopez	12,7	Brook Lopez	13,7
BOS	*Paul Pierce*	11,1	Jared Sullinger	5,9	Tyler Zeller*	7,2	Isaiah Thomas	13,9
CHA	Kemba Walker	11,2	Al Jefferson	15,4	Al Jefferson	9,1	Kemba Walker	14,2
CHI	*Joakim Noah*	9,1	*Joakim Noah*	13,3	*Pau Gasol*	15,0	Jimmy Butler	13,3
CLE	Kyrie Irving	10,7	Kyrie Irving	11,3	*LeBron James*	19,2	*LeBron James*	23,1
DAL	Vince Carter	7,7	Dirk Nowitzki	15,9	Tyson Chandler	10,8	Dirk Nowitzki	8,9
DEN	Kenneth Faried	9,0	*Kenneth Faried*	9,1	Ty Lawson	9,9	Nikola Jokic	9,5
DET	Greg Monroe	11,9	Andre Drummond	15,7	Andre Drummond	13,6	Andre Drummond	14,2
GS	Stephen Curry	15,3	Stephen Curry	18,6	Stephen Curry	22,2	Stephen Curry	27,6
HOU	James Harden	18,6	James Harden	18,0	*James Harden*	24,1	*James Harden*	23,1
IND	David West	10,5	*Paul George*	13,9	George Hill	6,6	*Paul George*	14,7
LAC	Chris Paul	17,9	Blake Griffin	17,8	Chris Paul	21,4	Chris Paul	18,4
LAL	*Kobe Bryant*	18,9	Pau Gasol	8,2	Ed Davis	7,8	Lou Williams	6,9
MEM	Marc Gasol	12,5	Mike Conley	11,0	Marc Gasol	14,9	Mike Conley	7,4
MIA	*LeBron James*	30,3	*LeBron James*	27,3	*Dwyane Wade*	10,7	Hassan Whiteside	16,0
MIL	Monta Ellis	8,1	John Henson	6,8	*Khris Middleton**	6,1	Greg Monroe	13,0
MIN	Nikola Pekovic	9,4	*Kevin Love*	21,5	Gorgui Dieng	7,3	*Karl-Anthony Towns*	15,7
NO	Anthony Davis	9,5	*Anthony Davis*	17,6	*Anthony Davis*	23,7	*Anthony Davis*	14,6
NY	*Carmelo Anthony*	17,7	*Carmelo Anthony*	20,8	*Carmelo Anthony*	7,9	*Carmelo Anthony*	12,4
OKC	*Kevin Durant*	27,7	*Kevin Durant*	30,1	Russell Westbrook	20,8	Russell Westbrook**	22,8
ORL	Nikola Vucevic	9,2	Nikola Vucevic	7,4	Nikola Vucevic	13,9	*Nikola Vucevic*	10,7
PHI	*Thaddeus Young*	10,2	*Thaddeus Young*	7,0	*Nerlens Noel*	5,1	Jahlil Okafor	5,2
PHX	Goran Dragic	8,4	Goran Dragic	14,5	Eric Bledsoe	10,4	Eric Bledsoe	4,8
POR	LaMarcus Aldridge	12,4	LaMarcus Aldridge	12,9	Damian Lillard	14,3	Damian Lillard	15,0
SA	*Tim Duncan*	13,4	Tim Duncan	10,6	Tim Duncan	12,4	*Kawhi Leonard*	18,5
SAC	*DeMarcus Cousins*	10,9	DeMarcus Cousins	17,8	*DeMarcus Cousins*	14,7	*DeMarcus Cousins*	14,6
TOR	Amir Johnson*	6,7	Kyle Lowry	13,1	Jonas Valanciunas	10,4	Kyle Lowry	15,9
UTAH	Al Jefferson	13,3	Derrick Favors	9,2	Gordon Hayward	12,7	Gordon Hayward	11,3
WSH	John Wall	7,9	John Wall	12,8	John Wall	12,6	John Wall	12,2
	Average star	12,9	Average star	14,0	Average star	13,0	Average star	14,2
	Average league	2,4	Average league	2,4	Average league	2,4	Average league	2,4
