# Supplementary material for: Exploring the impact of specialist and generalist stars on organizational performance
Source: PLoS One. 2026 May 28;21(5):e0349682. doi: 10.1371/journal.pone.0349682 (PMC13218541; doi:10.1371/journal.pone.0349682)
Supplement: S4 Table — 8,244 Observations. Relative performance is measured by the natural logarithm of the relative point differential (points scored/points allowed). Robust clustered standard errors by game (4,552 clusters) in parentheses. Significance levels are indicated as *** p < 0.01, ** p < 0.05, * p < 0.1. (PDF) [file pone.0349682.s007.pdf]

**Variables**	**(1)**	**(2)**	**(3)**	**(4)**	**(5)**	**(6)**
Generalist star		0.012***	0.012***	0.012***	0.012***	0.032***
		(0.003)	(0.003)	(0.003)	(0.004)	(0.005)
Generalist team			0.003	0.003	0.003	0.003
			(0.003)	(0.003)	(0.004)	(0.003)
Role switching				0.004	0.004	0.025***
				(0.003)	(0.003)	(0.005)
Generalist star x Generalist team					-0.000	
					(0.006)	
Generalist star x Role switching						-0.047***
						(0.009)
Average team salary	0.008***	0.008***	0.008***	0.008***	0.008***	0.007***
	(0.002)	(0.002)	(0.002)	(0.002)	(0.002)	(0.002)
Home game	0.056***	0.056***	0.056***	0.056***	0.056***	0.056***
	(0.004)	(0.004)	(0.004)	(0.004)	(0.004)	(0.004)
Team FEs	yes	yes	yes	yes	yes	yes
Opponent FEs	yes	yes	yes	yes	yes	yes
Season FEs	yes	yes	yes	yes	yes	yes
Constant	-0.026***	-0.032***	-0.033***	-0.035***	-0.035***	-0.056***
	(0.004)	(0.004)	(0.004)	(0.004)	(0.005)	(0.006)
R-squared	0.198	0.200	0.200	0.200	0.200	0.203
