# Supplementary material for: Exploring the impact of specialist and generalist stars on organizational performance
Source: PLoS One. 2026 May 28;21(5):e0349682. doi: 10.1371/journal.pone.0349682 (PMC13218541; doi:10.1371/journal.pone.0349682)
Supplement: S5 Table — Average marginal effects based on the interaction effects reported in model 6 in S4 Table. Relative performance is measured by the natural logarithm of the relative point differential (points scored/points allowed). Robust standard errors by game in parentheses. Significance levels are indicated as *** p < 0.01, ** p < 0.05, * p < 0.1. (PDF) [file pone.0349682.s008.pdf]

Role switching	Relative performance
Specialist star (season) $\rightarrow$ Specialist star (game)	-0.005***<br>(0.002)
Specialist star (season) $\rightarrow$ Generalist star (game)	0.020***<br>(0.004)
Generalist star (season) $\rightarrow$ Generalist star (game)	0.027***<br>(0.004)
Generalist star (season) $\rightarrow$ Specialist star (game)	0.005<br>(0.004)
