# Supplementary material for: Exploring the impact of specialist and generalist stars on organizational performance
Source: PLoS One. 2026 May 28;21(5):e0349682. doi: 10.1371/journal.pone.0349682 (PMC13218541; doi:10.1371/journal.pone.0349682)
Supplement: S7 Table — 5,841 Observations. Absolute performance is measured by the win-loss dummy. Relative performance is measured by the natural logarithm of the relative point differential (points scored/points allowed). Robust clustered standard errors by game (3,977 clusters) in parentheses. Significance levels are indicated as *** p < 0.01, ** p < 0.05, * p < 0.1. (PDF) [file pone.0349682.s010.pdf]

Variables	Absolute performance (1)	(2)	(3)	(4)	Relative performance (5)	(6)	(7)	(8)
Generalist star		0.113***	0.113***	0.113***		0.034***	0.034***	0.036***
		(0.019)	(0.020)	(0.022)		(0.005)	(0.005)	(0.006)
Generalist team			-0.002	-0.002			0.001	0.003
			(0.014)	(0.016)			(0.004)	(0.004)
Generalist star x Generalist team				-0.000				-0.007
				(0.029)				(0.008)
Average team salary	0.045***	0.040***	0.040***	0.040***	0.011***	0.009***	0.009***	0.009***
	(0.007)	(0.008)	(0.008)	(0.008)	(0.002)	(0.002)	(0.002)	(0.002)
Home game	0.180***	0.179***	0.179***	0.179***	0.056***	0.056***	0.056***	0.056***
	(0.015)	(0.015)	(0.015)	(0.015)	(0.004)	(0.004)	(0.004)	(0.004)
Team FEs	yes	yes	yes	yes	yes	yes	yes	yes
Opponent FEs	yes	yes	yes	yes	yes	yes	yes	yes
Season FEs	yes	yes	yes	yes	yes	yes	yes	yes
Constant	0.380***	0.267***	0.267***	0.267***	-0.033***	-0.067***	-0.068***	-0.069***
	(0.035)	(0.040)	(0.040)	(0.041)	(0.009)	(0.010)	(0.010)	(0.010)
R-squared	0.171	0.176	0.176	0.176	0.199	0.204	0.204	0.204
