# Supplementary material for: Exploring the impact of specialist and generalist stars on organizational performance
Source: PLoS One. 2026 May 28;21(5):e0349682. doi: 10.1371/journal.pone.0349682 (PMC13218541; doi:10.1371/journal.pone.0349682)
Supplement: S8 Table — As dependent variable serves the absolute performance (win-loss dummy). To discriminate between one-star and multiple-star teams, we first estimate the average EWA at the team level and then the EWA difference between the two players with the highest EWA in each team. If the EWA difference between the top two players is greater (smaller) than the average EWA at the team level, we consider these teams to be one-star (multiple-star) teams. We code absence only for injuries; suspensions, rest management, and personal reasons are excluded. EWA: Estimated Wins Added. Robust clustered standard errors by game in parentheses. Significance levels are indicated as *** p < 0.01, ** p < 0.05, * p < 0.1. (PDF) [file pone.0349682.s011.pdf]

**Variables**	**Full Sample**	**One-star teams**	**Multiple-star teams**
Star absence	-0.110***	-0.004	-0.149***
	(0.029)	(0.053)	(0.038)
Generalist star	0.088***	0.125***	0.017
	(0.015)	(0.021)	(0.031)
Star absence x Generalist star	-0.042	-0.161**	0.144
	(0.047)	(0.067)	(0.107)
Generalist team	0.002	-0.004	0.004
	(0.011)	(0.014)	(0.017)
Average team salary	0.043***	0.029***	0.057***
	(0.006)	(0.008)	(0.011)
Home game	0.179***	0.182***	0.174***
	(0.013)	(0.015)	(0.018)
Team FEs	yes	yes	yes
Opponent FEs	yes	yes	yes
Season FEs	yes	yes	yes
Constant	0.338***	0.197***	0.466***
	(0.017)	(0.044)	(0.062)
Observations	9,214	5,529	3,685
R-squared	0.173	0.178	0.189
