# Supplementary material for: Exploring the impact of specialist and generalist stars on organizational performance
Source: PLoS One. 2026 May 28;21(5):e0349682. doi: 10.1371/journal.pone.0349682 (PMC13218541; doi:10.1371/journal.pone.0349682)
Supplement: S9 Table — To identify multiple-star teams, we first estimate the average EWA at the team level and then the EWA difference between the two players with the highest EWA in each team. If the EWA difference between the top two players is greater (smaller) than the average EWA at the team level, we consider these teams to be one-star (multiple-star) teams. EWA: Estimated Wins Added. (PDF) [file pone.0349682.s012.pdf]

**2013**	**2014**	**2015**	**2016**
BKN	BKN	ATL	ATL
CHI	BOS	BOS	CHI
DAL	DEN	CHI	DEN
DEN	LAC	DAL	MEM
IND	MEM	IND	MIL
LAC	MIL	MIA	OKC
MEM	ORL	MIL	PHI
MIL	PHI	MIN	PHX
NO	POR	PHX	TOR
PHI	SA	POR	UTAH
POR	TOR	SA	
SA	UTAH	TOR	
TOR		UTAH	
