# Supplementary material for: Exploring the impact of specialist and generalist stars on organizational performance
Source: PLoS One. 2026 May 28;21(5):e0349682. doi: 10.1371/journal.pone.0349682 (PMC13218541; doi:10.1371/journal.pone.0349682)
Supplement: S1 File — (PDF) [file pone.0349682.s014.pdf]

In equilibrium, the offensive team will choose play types p with higher skill S_p with higher probability, while the defensive team will put more weight on defending against those play types. While the “strong” play types are observed more often than the weaker ones, the expected scoring probability in equilibrium will be identical for all play types: In any equilibrium in mixed strategies, the strategy of the defense, δ, must make the offense indifferent between its options; otherwise, the offense would not mix. The payoff of the team playing offense is the scoring probability $P(S_p, D, \delta_p)$, while the payoff for the defensive team is exactly the counter-probability. Thus, in any mixed strategy equilibrium, the offensive team is willing to mix among these strategies if the scoring probability is the same for all offensive play types (given δ). Therefore,

$$\varphi(S_1, D, \delta_1) = \varphi(S_2, D, \delta_2) = ... = \varphi(S_P, D, \delta_P). \tag{1}$$

The defensive team allocates the defensive weights δ such that the marginal effect of an increase in δ_p on the scoring probability is the same for all P play types. If this was not true, the defensive team could improve its expected payoff (that is, reduce the expected scoring probability) by allocating more weight to defending the play type with the higher marginal effect of δ and less weight to the play type(s) with a lower marginal effect. Therefore,

$$\frac{\partial \sigma_1 \varphi(S_1, D, \delta_1)}{\partial \delta_1} = \frac{\partial \sigma_2 \varphi(S_2, D, \delta_2)}{\partial \delta_2} = ... = \frac{\partial \sigma_P \varphi(S_P, D, \delta_P)}{\partial \delta_P} \tag{2}$$

Eq (1) and Eq (2) each contain $P - 1$ equations. Together with the property that all weights for offensive weights σ_1, σ_2..., and all weights for defensive weights δ_1, δ_2..., must add up to 1, we have $2 \cdot (P - 1) + 2 = 2P$ equations that we can use to determine the $2P$ unknowns that pin down the offensive strategy σ and the defensive strategy δ.
