# Supplementary material for: Exploring the impact of specialist and generalist stars on organizational performance
Source: PLoS One. 2026 May 28;21(5):e0349682. doi: 10.1371/journal.pone.0349682 (PMC13218541; doi:10.1371/journal.pone.0349682)
Supplement: S2 File — (PDF) [file pone.0349682.s015.pdf]

First, we assume that the probability that a particular player m is the player who finishes a particular play type p, $\omega_{m,p}$, is positively correlated with his individual skill $s_{m,p}$ relative to the individual skills of his teammates $s_{t,p}$ for this play type. In particular, we assume

$$\omega_{m,p} = \frac{s_{m,p}}{\sum_{n=1}^{5} s_{m,p}} \tag{1}$$

Then $w_{m,p}$, the weight (or share) of a particular play type p of a particular player m is

$$w_{m,p} = \frac{\sigma_p \omega_{m,p}}{\sum_{p=1}^{P} \sigma_p \omega_{m,p}} \text{ with} \tag{2}$$

$$\sum_{p=1}^{P} w_{m,p} = 1 \tag{3}$$
